# Spotlight on Zebrafish: Translational Impact

**DOI:** 10.1242/dmm.017004

**Published:** 2014-07

**Authors:** E. Elizabeth Patton, Paraminder Dhillon, James F. Amatruda, Lalita Ramakrishnan

**Affiliations:** 1 MRC Institute of Genetics and Molecular Medicine, University of Edinburgh, UK.; 2 Disease Models & Mechanisms, The Company of Biologists, Bidder Building, 140 Cowley Road, Cambridgeshire, UK.; 3 UT Southwestern Medical Center, Dallas, TX, USA.; 4 University of Washington, Seattle, Washington, USA.

## Abstract

In recent years, the zebrafish has emerged as an increasingly prominent model in biomedical research. To showcase the translational impact of the model across multiple disease areas, *Disease Models & Mechanisms* has compiled a Special Issue that includes thought-provoking reviews, original research reporting new and important insights into disease mechanisms, and novel resources that expand the zebrafish toolkit. This Editorial provides a summary of the issue’s contents, highlighting the diversity of zebrafish disease models and their clinical applications.

**Figure:**
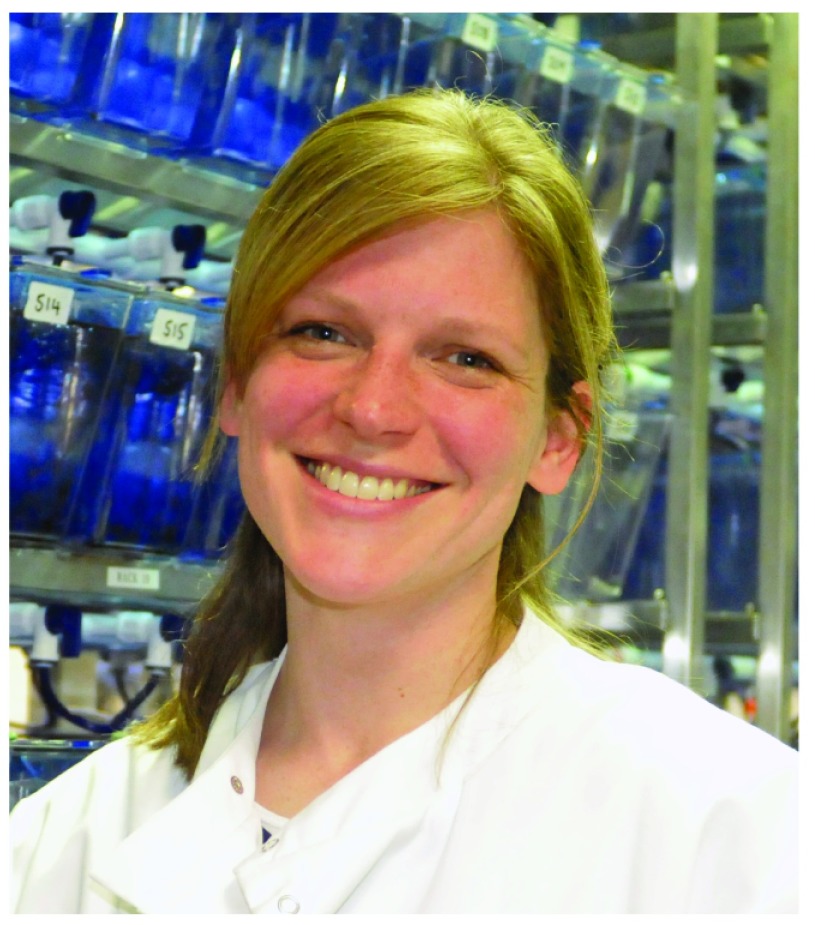
E. Elizabeth Patton

**Figure:**
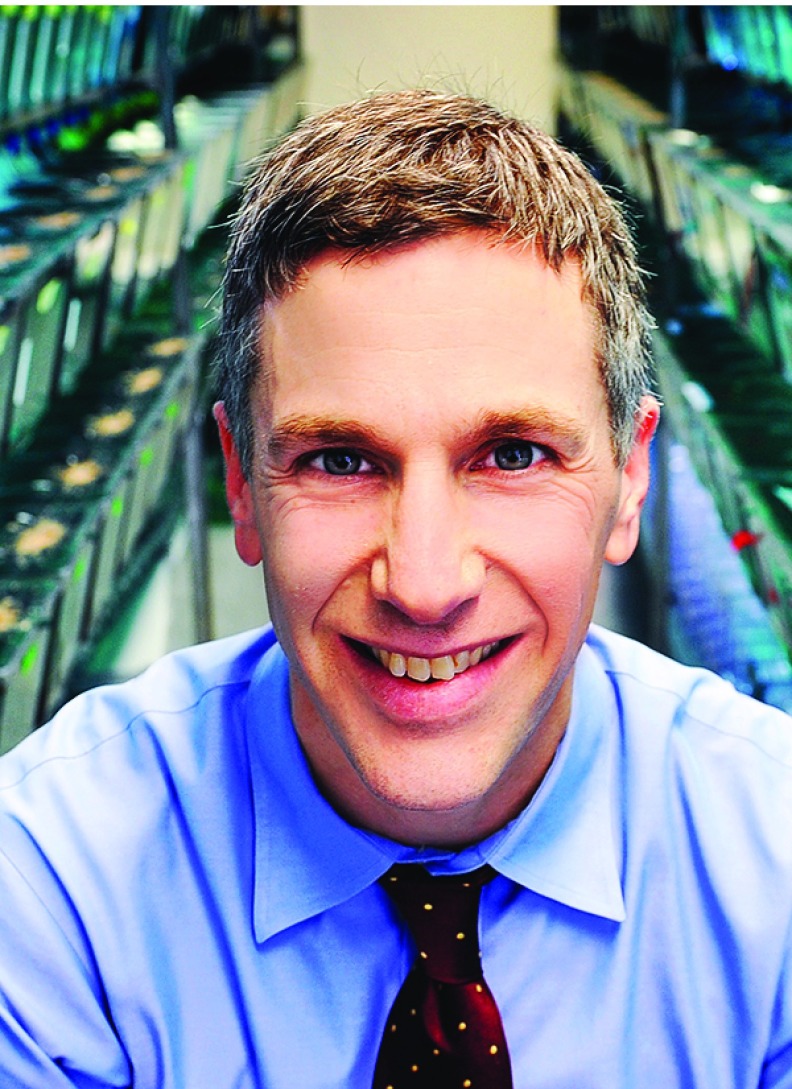
James F. Amatruda

**Figure:**
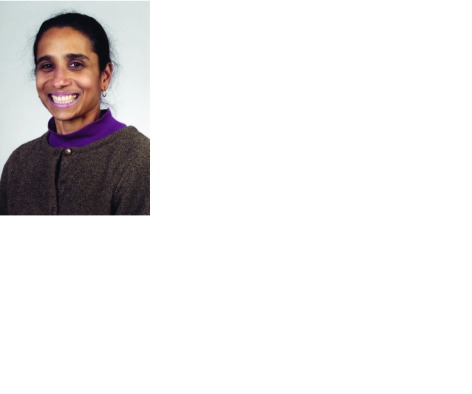
Lalita Ramakrishnan

## Special Issue: Spotlight on Zebrafish

One aim of a *Disease Models & Mechanisms* (DMM) ‘Special Issue’ is to highlight how emerging disease models can lead to exceptional growth in particular areas of translational research. This is especially true for this issue, Spotlight on Zebrafish: Translational Impact. The zebrafish has traditionally been used to study developmental biology. Its optical transparency for the first few weeks, high fecundity and *ex vivo* fertilization have meant that the fundamental processes and mechanisms of vertebrate embryo development from a single cell through to a swimming fish can be studied in exquisite detail. Over the past decade these same features have enabled the zebrafish to become a preeminent disease model and tool for studying disease mechanisms. Importantly, discoveries in zebrafish disease models are leading to new perspectives on human disease and new drugs that are entering the clinic in diverse areas from cancer to tuberculosis.

We are delighted to present an issue packed with reviews, research and resource articles from researchers at the cutting-edge of their respective disease area of interest. The issue also includes a compelling interview with **Len Zon**, pioneer in the zebrafish disease models community, and a unique poster representation of the translational applications of zebrafish research. Here, we summarize the contents of the issue, and give our views on what makes each article special.

## Front section of the issue: Reviews and more

Kicking off the front section of the issue, **Jennifer Phillips and Monte Westerfield** provide an overview of the use of zebrafish in translational research in an ‘At a Glance’ poster article. The poster, which can be downloaded from the DMM website, highlights the unique strengths of the model and the advances in technologies that have expanded the zebrafish toolbox. The authors also illustrate recent examples of successful clinical translation of zebrafish studies. One of the examples, the groundbreaking discovery by the Zon lab of a new treatment strategy for melanoma, is described by **Len Zon** in his own words in an exclusive ‘Model for Life’ interview. As well as telling the stories behind some of the key milestones in his career, Len provides his perspectives on emerging challenges and opportunities in zebrafish research.

In the first of two cancer-focused Reviews, **Jason Berman and colleagues** provide a compelling argument to support the use of zebrafish xenotransplantation for preclinical cancer drug screening and testing. Next, **Jessica Blackburn and David Langenau** explain how intratumoral heterogeneity can present an obstacle to cancer therapy, and underscore the utility of zebrafish models for exploring the molecular basis of clonal evolution in tumorigenesis.

**Aarti Asnani and Randal Peterson** then review the impressive array of zebrafish studies that have provided important mechanistic insights into cardiovascular disorders and paved the way for development of novel therapies. **Wolfram Goessling and Trista North** also touch upon cardiac regeneration as part of a broader review of recent zebrafish-driven advances in regenerative medicine. Focusing particularly on the hematovascular and gastrointestinal systems, the authors discuss how the regenerative capacity of the organism can be exploited to devise therapeutic strategies to promote organ-specific repair in response to injury and disease.

An important area that has advanced significantly as a direct result of using the zebrafish is tuberculosis research, and these discoveries and their therapeutic implications are reviewed by **Mark Cronan and David Tobin**. A Review by **Annemarie Meijer and colleagues** further illuminates the power of the model for understanding and treating infectious diseases. Their article describes the insights into macrophage-pathogen interactions that have been gained using zebrafish infection models, and illustrates how these insights have unveiled promising therapeutic candidates to combat diverse bacterial and fungal pathogens.

Finally, **Pierre Drapeau’s group** review zebrafish models of motor neuron disorders, with a particular focus on amyotrophic lateral sclerosis (ALS). They highlight how these models have enhanced our understanding of the underlying disease pathology and are posed to advance drug discovery in the near future.

## New research, models and tools

In the research section of the issue, a striking example of how the zebrafish can enable new insight into disease processes is presented in an elegant study by **Alexander Apschner, Stefan Schulte-Merker and colleagues**. Through phenotypic analysis of a genetic mutant in ectonucleoside pyrophosphate/phosphor-diesterase (*enpp1*), they discover ectopic soft-tissue calcifications, as observed in humans with *ENPP1* mutations. Interestingly, there is premature development of osteoclast-like cells at the ectopic mineralization sites, suggesting that osteoclasts might be promoting regression at mineralization sites. This is a new concept for ectopic mineralization diseases, such as generalized arterial calcification of infancy and pseudoxanthoma elasticum, and has implications for bisphosphonate treatments that both inhibit progression of calcifications but also have known inhibitory effects against osteoclasts.

The high genomic conservation between zebrafish and humans, including the presence of orthologs for over 70% of human genes, enables discoveries in zebrafish to be directly tested for relevance in human disease. **Ana Vacaru, Kirsten Sadler and colleagues** analyze zebrafish liver steatosis, protein markers and target gene expression to provide a new perspective on the unfolded protein response (UPR) and its role in fatty liver disease (FLD). They discover that, in response to endoplastic reticulum (ER) stress, zebrafish embryos have three distinguishable gene expression responses, and only one of these responses leads to FLD (‘stressed UPR’). Importantly, markers of this stressed UPR significantly correlated with steatosis in zebrafish, and also in humans with FLD. Surprisingly, a different UPR subclass leads to an adaptive and protective response, suggesting that promoting an adaptive UPR response might actually be beneficial in the context of diseases caused by high UPR induction.

Gene expression analysis is also an important feature in a paper that aims to dissect the molecular mechanisms underlying a pancreatic phenotype in a *urod* mutant, a model of human hepatoerythropoietic porphyria. By analyzing the gene expression changes in *urod* mutants, **Shuqing Zhang, Han Wang and colleagues** discover a class of peptidase precursor genes that are specifically downregulated in the mutant zebrafish. The authors go on to demonstrate a heme-dependent mechanism that regulates expression of exocrine zymogens by enabling a switch from a repressive Bach1b-MafK heterodimer to an activating Nrf2a-MafK heterodimer. This heme-dependent mechanism and decreased pancreatic zymogen production could explain some of the clinical features of porphyria, which include acute episodic abdominal pain.

Forward genetic screens have been a long-standing feature and strength of the zebrafish system. **Tamara Stawicki, David Raible and colleagues** use forward genetics to identify new genes involved in resistance to hearing loss, a disorder that affects over 35% of people over 65. They identify a mutation in the transcription factor *gcm2* that is resistant to neomycin-induced hair cell death, and is important for whole body pH regulation. Defects in *gcm2* lead to impaired mechanotransduction ability, subsequent reduced drug uptake and audiovestibular behavioral defects. This is the second example of a genetic mutation that links pH regulation to hair cell sensitivity to ototoxic drugs, and points to pH regulation as an important role in this process.

Zebrafish models can fill the gap for diseases where there are few models or treatments. **Alexa Burger, Daniel Haber and colleagues** demonstrate this by providing a new model of chordoma, a rare type of bone cancer that is thought to arise from the remnants of embryonic notochord cells. Until now, there has been no animal model of chordoma, few cell lines and limited treatment options. Burger et al. show that expression of oncogenic *HRAS*^V12^ in the notochord rapidly induces the development of tumors that resemble human chordoma. Treatment with the mTOR inhibitor rapamycin, which has shown promise for treating human chordoma, delays tumor onset and improves zebrafish survival. This model opens new doors for high-throughput chemical screening for therapeutic agents to treat this cancer.

The development of new and improved disease models depends on good tools and methods, and two studies presented here should greatly enhance the zebrafish toolkit. **Rosa Miyares, Vitor de Rezende and Steven Farber** present zebrafish yolk lipid processing as a new tool to study lipid metabolism and dyslipidemias, a major risk factor for heart disease and cause of mortality. Using fatty acids labeled with fluorescent or radioactive tracers, the metabolism and fate of the lipid is followed in live zebrafish. The methods described will be important for studying the fundamental processes of lipid metabolism, and also for studying lipid metabolism in the context of genetic models of disease and developing new therapeutic regulators. **Wouter Koole and Marcel Tijsterman** use a genetic approach that relies on microsatellite instability to develop a new tool for mosaic analysis. Optimized for the GAL4-UAS system, the authors show that placing genes behind unstable microsatellite-dependent sequences can lead to stochastic activation *in vivo*. They demonstrate the success of their approach by placing oncogenic *H-RAS*^G12V^ under the control of microsatellite sequences and demonstrate tumor development from a single cell. This approach should enable the etiology and pathology of cancer to be studied within the context of otherwise wild-type animals.

One of the principal strengths of the zebrafish is its amenability to intravital imaging of cellular processes in unprecedented detail. **Tjakko Van Ham, Ben Giepmans and colleagues** use imaging to study the resolution of neuroinflammation *in vivo*. Using a model of neurodegeneration that genetically targets brain cells for cell death, they follow the kinetics and nature of the immune response following injury and find that peripheral macrophages and resident microglia clear the dying cells. The timing of these cells is different and, remarkably, clearance involves phagocyte apoptosis and engulfment by microglia. These new insights into the cellular mechanisms involved in clearing degenerated cells could be important for diseases such as Alzheimer’s disease. Exquisite imaging is also a feature of the research by **Mai Nguyen-Chi, Georges Lutfalla and colleagues**, who were able to model chronic inflammation using a transient *Escherichia coli* infection model in the notochord. Bone and cartilage inflammation can lead to progressive bone and joint destruction, but models of chronic inflammation in these tissues are lacking. Using the notochord as a site for inflammation, the authors transiently infect the notochord with an *E. coli* pathogen that is rapidly cleared but nonetheless triggers a persistent immune response. The persistent immune response is characterized by neutrophils undergoing degranulation next to the notochord in the absence of a pathogen *in vivo*, leading to tissue damage and eventually dysmorphic vertebrae development. Thus, both these studies demonstrate the power of high-resolution live imaging in zebrafish models to visualize novel cellular events that underlie pathology.

Imaging is not just limited to the embryonic forms. Using larval and adult zebrafish, **Marco Schiavone, Francesco Argenton and colleagues** engineer reporter lines for signaling pathways into a model of pancreatic adenocarcinoma and medulloblastoma. This enables the dynamics of cancer signaling pathways to be traced *in vivo*, and reveals tissue-specific regulation of cell-signaling pathways between cancer types. These lines prove valuable for visualizing tumor development *in situ*, and could become particularly relevant in the context of visualizing cancer drug response in live adult fish.

Ultimately, understanding disease mechanism in zebrafish disease models can lead to new treatments. This is exemplified in a study by **Nadia Danilova, Shuo Lin and colleagues**, who find activation of DNA damage checkpoints, and upregulation of genes involved in nucleotide catabolism and biosynthesis, in two genetic models of ribosomal protein (RP) deficiency. Human fetal liver cells deficient for RPS19 also show signs of replicative stress and the DNA damage response, suggesting that the cellular response to RP deficiency is conserved between zebrafish and humans. Because nucleosides have been used to treat other conditions of replicative stress in cells, the authors treat their RP-deficient zebrafish models with exogenous nucleosides and discover that this leads to improved survival, increased blood count and decreased morphological defects. The authors suggest that exogenous nucleoside supplements might be of novel therapeutic benefit for humans with RP deficiency, as observed in Diamond Blackfan anemia patients.

What is particularly impressive about our collection – and the field at large – is the diversity of disease models and their relevance to human disease. The response to our ‘call for papers’ and invitations to write reviews has been outstanding, and we hope you enjoy reading and sharing the issue as much as we have enjoyed bringing it to you. Please be sure to keep an eye on the DMM zebrafish collection page at http://dmm.biologists.org/site/collections/zebrafish.xhtml to read further zebrafish articles reporting new and exciting insights into disease in the coming months.
